# rAAV-Mediated Subcellular Targeting of Optogenetic Tools in Retinal Ganglion Cells *In Vivo*

**DOI:** 10.1371/journal.pone.0066332

**Published:** 2013-06-14

**Authors:** Chaowen Wu, Elena Ivanova, Yi Zhang, Zhuo-Hua Pan

**Affiliations:** 1 Department of Anatomy/Cell Biology, Wayne State University School of Medicine, Detroit, Michigan, United States of America; 2 Department of Ophthalmology, Wayne State University School of Medicine, Detroit, Michigan, United States of America; Oregon Health & Science University, United States of America

## Abstract

Expression of optogenetic tools in surviving inner retinal neurons to impart retinal light sensitivity has been a new strategy for restoring vision after photoreceptor degeneration. One potential approach for restoring retinal light sensitivity after photoreceptor degeneration is to express optogenetic tools in retinal ganglion cells (RGCs). For this approach, restoration of ON and OFF center-surround receptive fields in RGCs, a key feature of visual information processing, may be important. A possible solution is to differentially express depolarizing and hyperpolarizing optogenetic tools, such as channelrhodopsin-2 and halorhodopsin, to the center and peripheral regions of the RGC dendritic field by using protein targeting motifs. Recombinant adeno-associated virus (rAAV) vectors have proven to be a powerful vehicle for in vitro and in vivo gene delivery, including in the retina. Therefore, the search for protein targeting motifs that can achieve rAAV-mediated subcellular targeted expression would be particularly valuable for developing therapeutic applications. In this study, we identified two protein motifs that are suitable for rAAV-mediated subcellular targeting for generating center-surround receptive fields while reducing the axonal expression in RGCs. Resulting morphological dendritic field and physiological response field by center-targeting were significantly smaller than those produced by surround-targeting. rAAV motif-mediated protein targeting could also be a valuable tool for studying physiological function and clinical applications in other areas of the central nervous system.

## Introduction

The severe loss of photoreceptor cells in inherited and acquired retinal degenerative diseases, such as retinitis pigmentosa, could result in partial or complete blindness. Expression of optogenetic tools in surviving inner retinal neurons to impart retinal light sensitivity has been a new strategy for restoring vision after photoreceptor degeneration [1]–[7]. One approach is to express microbial rhodopsins in retinal ganglion cells (RGCs). ON and OFF light responses can be restored in RGCs by expressing channelrhodopsin-2 (ChR2) and halorhodopsin (NpHR), respectively [1], [8]. However, by directly rendering RGCs photosensitive and bypassing normal intraretinal processing, pathways that lead to the formation of center-surround receptive fields, a key feature of visual processing in the retina for enhancing spatial contrast sensitivity [9], are lost. A possible solution to a create center-surround receptive field is to differentially express depolarizing and hyperpolarizing optogenetic tools to the center and peripheral regions of the RGC dendritic field by using protein targeting motifs [10].

Motifs are an important mechanism for the localization of functional membrane proteins, such as membrane channels and receptors, to specific subcellular regions [11]. A number of subcellular protein targeting motifs have been reported to show polarized expression in mammalian neurons [12]–[20]. However, most of these studies were carried out in cultured pyramidal neurons of the hippocampus or cortex. Differential expression of ChR2 and NpHR using ankyrin-G and postsynaptic density (PSD) motifs has been shown to generate a center-surround receptive field in RGCs [10]; but this was only achieved by biolistic particle delivery. Motifs that can achieve subcellular targeting with an in vivo delivery system are particularly valuable for developing potential therapeutic applications.

Recombinant adeno-associated virus (rAAV) vectors have proven to be a promising vehicle for in vivo gene delivery for therapeutic applications because of their nonpathogenic and nonimmunogenic properties towards the host, efficient transduction rate in both dividing and non-dividing cells, and broad cell and tissue tropisms [21], [22]. rAAV2 is the best characterized viral vector serotype and has currently been used in clinical trials in ocular gene therapy [23], [24]. Thus, the development of rAAV-mediated subcellular targeting of optogenetic tools in RGCs is a rational approach. However, whether the motifs reported to have achieved polarized expression in other systems could also work in the rAAV mediated delivery system, especially in RGCs, remains unknown.

In this study, we examined the suitability of eight motifs for recreating the center-surround antagonistic receptive field through rAAV-mediated delivery. All of these motifs have been previously reported to produce polarized gene expression in vitro or in vivo in hippocampal or cortical neurons. Four of the motifs were found to show polarized expression in RGCs. Based on morphological analysis and physiological recordings, we identified two motifs that could be suitable for generating center-surround receptive fields in RGCs.

## Materials and Methods

### Viral Constructs

rAAV2 vectors carrying a CAG (a hybrid CMV early enhancer/chicken β-actin) promoter and a fusion construct of channelrhodopsin-2 and GFP (ChR2-GFP) was modified from a previously reported construct [1] by inserting motif sequences at the 3′ end of GFP. The virus vectors were packaged at the Gene Transfer Vector Core of the University of Iowa. rAAV2 constructs carrying an elongation factor I alpha (EF1α) promoter and a double-floxed inverted open-reading frame (DIO) sequence encoding ChR2-YFP or NpHR-YFP were modified from a construct obtained from Dr. Karl Deisseroth [25] by replacing YFP with mCherry or inserting motif sequences at the 3′ end of YFP. The DIO virus vectors were packaged and affinity purified at the virus core facility of University of Pennsylvania.

### Animals and Virus Injection

All animal experiments and procedures were approved by the Institutional Animal Care Committee at Wayne State University, and were in accord with the NIH Guide for the Care and Use of Laboratory Animals. Adult C57BL/6J mice or Tg(Pcp2-cre)1Amc/J (referred to as Pcp2-cre) transgenic mice aged 1–2 months were used for virus injections. Animals were anesthetized by intraperitoneal injection of ketamine (120 mg/kg) and xylazine (15 mg/kg). Under a dissection microscope, ∼1.0 µl of viral vector suspension was injected into the intravitreal space of each eye using a Hamilton syringe with a 32-gauge blunt-ended needle. Mice received viral vector injections at 1–4×10^12^ GP/ml. Animals were used for experiments at least one month after viral injection.

### Fluorescence Profile and Dendritic Field Measurements

Animals were sacrificed by CO_2_ asphyxiation followed by decapitation and enucleation. Enucleated eyes were fixed in 4% paraformaldehyde in phosphate buffer (PB) at room temperature (RT) for 20 minutes. Fluorescence expression was examined in flat-mounted retinas. All images were acquired using a Zeiss Axioplan 2 microscope with Apotome (Carl Zeiss) with the AxioVision software. Z-stack images were taken at ∼560 ms exposure time at optical sections of 1 µm apart to capture the axon, soma, and the entire depth of the dendritic tree of each RGC. Images were exported for fluorescence intensity comparisons. Fluorescence intensity (FI) profiles were measured using the software ImageJ (obtained from NIH) by applying 5-pixel wide lines perpendicular to the cell membrane and averaging the peak FI measurements at the membrane. ImageJ fluorescence scale ranges from 0–225 where 0 corresponds to no fluorescence (black) and 225 correspond to complete saturation (white). For each RGC, soma FI profile was obtained by averaging 3 measurements, dendrite FI profiles were obtained by averaging 9 measurements (3 proximal, 3 intermediate, and 3 distal dendrite measurements), and axon FI profile was obtained by averaging 3 measurements beyond the axon initial segment (AIS). RGC morphological dendritic field sizes were assessed by approximating the GFP-positive dendritic tree area using the AxioVision software by outlining the outermost points of observed fluorescence, rendering the outlined area into a circle then determining the diameter of that circle. Initial measurements were made in pixels which were converted into µm measurements.

### Multi-electrode Array (MEA) Recordings

MEA recordings were based on procedures previously reported [26]. Briefly, the dissected retina was mounted photoreceptor side down on a piece of nitrocellulose filter paper (Millipore Corp., Bedford, MA). The mounted retina was placed in the MEA-60 multielectrode array recording chamber with the RGC layer touching the 10 µm diameter electrodes spaced 200 µm apart (Multi Channel System MCS GmbH, Reutlingen, Germany). During experiments, the retina was continuously perfused in 34°C oxygenated extracellular solution containing (in mM): NaCl, 124; KCl, 2.5; CaCl_2_, 2; MgCl_2_, 2; NaH_2_PO_4_, 1.25; NaHCO_3_, 26; and glucose, 22 (pH 7.35 with 95% O_2_ and 5% CO_2_). Recordings began approximately 60 min after placing the retina in the multi-electrode array recording chamber. Signals were filtered between 200 Hz (low cut off) and 20 kHz (high cut off) and recorded by MC Rack software (Multi Channel Systems).

### Light Stimulation

For MEA recordings, light stimuli were generated by a modified and laser-based LCD projector (8500, Espson). A 200 mW blue laser (473 nm) and a 300 mW green laser (532 nm) (Changchun New Industries Optoelectronics Tech. Co., Ltd., China) were coupled via optical fiber to a projector which projected the stimuli to the bottom of the MEA recording chamber. The projector rendered the 800×600 pixel (px) computer generated stimulus field into a 8×6 mm light stimulus field, therefore a 1 px corresponded to ∼10 µm. The light intensity is 6×10^15^ and 1×10^16^ photon/cm^2^ sec for blue (473 nm) and green (532 nm) laser, respectively. Custom light pattern stimulation programs were designed in the Neurophysiology (Vision Research Graphics, Inc., Durham, NH, USA) software. The full-field program consisted of a 800×600 px stimulus. The stepping bar program consisted of a 200×6000 µm bar stimulus stepping at 20 µm increments. All light stimulation patterns were presented for 1 s followed by a 9 s inter-trial interval.

### Physiological Response Field Measurements

MEA responses from individual neurons were analyzed using the Offline Sorter software (Plexon, Inc., Dallas, TX). The total number of steps that elicited ChR2- or NpHR-mediated spiking activity was determined and then RGC response field size was multiplied by 20 µm/1 step to convert the response field into µm measurements for comparisons between groups.

#### Statistical analysis

Morphological and physiological receptive field sizes are presented as mean ± standard error. The Mann-Whitney test with post-hoc bonferroni correction was used for the independent samples comparisons between motif-targeted groups and the control for morphological and physiological receptive field size.

## Results

### In vivo Motif-targeted ChR2-GFP Expression in RGCs

We examined eight motifs for rAAV2-mediated expression of ChR2-GFP in RGCs in vivo. Two were potential center targeting motifs, Kv2.1-motif [12] and Nav1.6-motif [13], previously reported to target the soma/proximal dendrites and axon initial segment (AIS) of a neuron, respectively. The remaining were potential surround targeting motifs, AMPAR-motif [14], Kv4.2-motif [15], MLPH-motif [16], [17], nAChR-motif [18], NLG1-motif [19], and TLCN-motif [20], and all were previously reported to target the somatodendritic region of a neuron in vitro or in vivo. All motifs are summarized in Table 1.

**Table 1:** Summary of the targeting motifs examined in this study: Motif notation, origin protein, binding domain, amino acid sequences, and reported subcellular targeted site.

Motif notation	Origin protein	Binding domain	Amino acid sequence (3′–5′)	Targeted site
Kv2.1	voltage-gated K^+^ channel 2.1	Cytoplasmic C-terminal	QSQPILNTKEMAPQSKPPEELEMSSMPSPVAPLPARTEGVIDMRSMSSIDSFISCATDFPEATRF	Soma and proximal dendrites
Nav1.6	Voltage-gated Na^+^ channel 1.6	Ankyrin binding domain	TVRVPIAVGESDFENLNTEDVSSESDP	Axon initial segment
AMPAR	AMPAR GluR1 subunit	Cytoplasmic C-terminal	EFCYKSRSESKRMKGFCLIPQQSINEAIRTSTLPRNSGA	Somatodendritic
Kv4.2	Voltage-gated K^+^ channel 4.2	Dileucine	FEQQHHHLLHCLEKTT	Somatodendritic
MLPH	Melanophilin	Myosin binding domain	RDQPLNSKKKKRLLSFRDVDFEEDSD	Somatodendritic
nAChR	nAChR α7 subunit	Tyrosine-Dileucine	GEDKVRPACQHKPRRCSLASVELSAGAGPPTSNGNLLYIGFRGLEGM	Somatodendritic
NLG1	Neuroligin-1	Cytoplasmic C-terminal	VVLRTACPPDYTLAMRRSPDDVPLMTPNTITM	Somatodendritic
TLCN	Telencephalin	Phenylalanine-based	QSTACKKGEYNVQEAESSGEAVCLNGAGGGAGGAAGAEGGPEAAGGAAESPAEGEVFAIQLTSA	Somatodendritic

**Figure 1:**
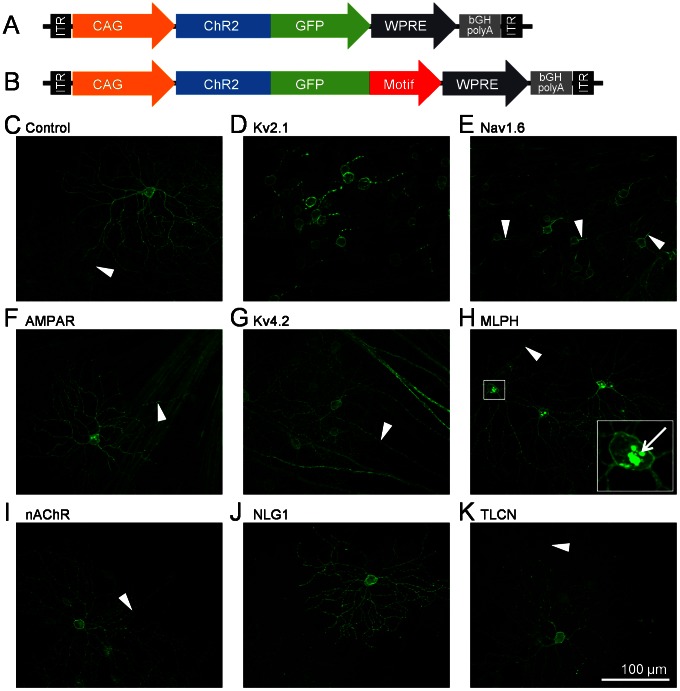
Viral constructs and expression in RGCs. A, B) The control AAV2/2 viral construct carrying the ChR2-GFP gene (A) and the modified construct with targeting-motif inserted in the 3′ end of GFP (B) are driven by the ubiquitous CAG promoter. C–K) RGC expression of the ChR2-GFP (C) without motif targeting (control), with (D) Kv2.1-motif targeting, (E) Nav1.6-motif targeting, (F) AMPAR-motif targeting, (F) Kv4.2-motif targeting, (H) nAchR-motif targeting, (I) MLPH-motif targeting where soma inclusions are indicated by an arrow in the inset high magnification image, (J) NLG1-motif targeting, and (K) TLCN-motif targeting. RGC axons are indicated by arrowheads.

In vivo expression in RGCs was first achieved by intravitreal injection of rAAV2 vectors carrying the ChR2-GFP without motif as control (Fig. 1A) and with the motif sequences inserted at the 3′ end of GFP (Fig. 1B). In retinas injected with the non-motif control construct, ChR2-GFP expression was observed on membrane surfaces of RGC somas, dendrites, and axons (indicated by arrowheads) (Fig. 1C). In retinas injected with the Kv2.1- and Nav1.6-motifs, the expression of ChR2-GFP was markedly different from that of the control. In the Kv2.1-motif injected retinas, the expression was mainly located on the membrane surface of RGC soma, axon initial segment, and in some instances on the proximal dendrites (Fig. 1D). In the Nav1.6-motif injected retinas, the expression was localized mainly on the membrane surface of the RGC soma, axon initial segment, and displayed a graded decrease in dendritic expression from proximal to distal dendrites (Fig. 1E). In both cases, the expression in distal axons was significantly reduced. In retinas injected with the somatodendritic motifs, the expression was observed in RGC somas and dendrites (Fig. 1F–K). The axonal expression of AMPAR-, Kv4.2-, nAChR-, and TLCN-motif targeting (indicated by arrowheads) was not found to differ from the control, but was significantly reduced with the MLPH and NLG1 motifs. In the MLPH-motif targeted RGCs, intracellular inclusions were consistently observed in the soma (Fig. 1H; marked by an arrow in the inset high magnification image).

**Figure 2:**
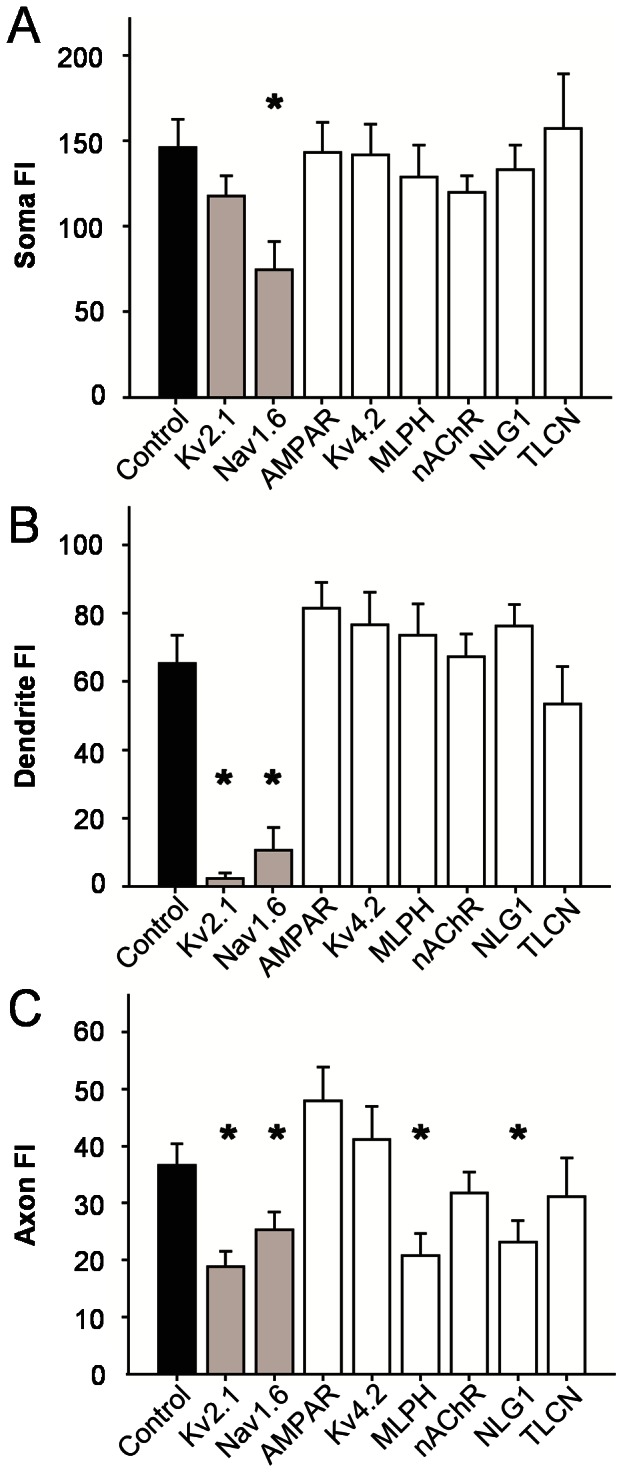
Fluorescence intensity (FI) comparisons between control and motif-targeted RGCs. GFP-FI profiles obtained from soma, dendrites, and axon of motif-targeted RGCs were compared to the non-targeted control expression (*p<0.001). A) Soma FIs for each motif-targeted group are as follows (mean ± SEM): Control: 146±8; Kv2.1∶118±6; Nav1.6∶75±8; AMPAR: 143±9; Kv4.2∶142±9; MLPH: 129±9; nAchR: 120±5; NLG1∶133±7; TLCN: 157±16. Soma FI of Nav1.6-motif targeted expression was significantly different from control. B) Dendrite FIs for each motif-targeted group are as follows: Control: 65±4; Kv2.1∶2±1; Nav1.6∶11±3; AMPAR: 82±4; Kv4.2∶77±5; MLPH: 74±5; nAchR: 67±3; NLG1∶76±3; TLCN: 53±6. Dendrite FI of Kv2.1- and Nav1.6-motif targeted expression was significantly different from control. C) Axon FIs for each motif-targeted group are as follows: Control: 37±2; Kv2.1∶19±1; Nav1.6∶25±2; AMPAR: 48±3; Kv4.2∶41±3; MLPH: 21±2; nAchR: 32±2; NLG1∶23±2; TLCN: 31±3. Axon FI of Kv2.1-, Nav1.6-, MLPH-, and NLG1-motif targeted expression was significantly different from control. Number of RGCs analyzed in each group are given in parentheses: Control (29), Kv2.1 (24), Nav1.6 (24), AMPAR (23), Kv4.2 (26), MLPH (25), nAchR (29), NLG1 (25), TLCN (19).

Next we quantitatively measured the degree of expression polarization in RGCs. For comparison, all fluorescence images were obtained at similar exposure times. Fluorescence intensity (FI) profiles taken from the soma, dendrites, and axons (beyond the AIS, see methods) were directly compared between the motif-targeted groups and the control group (Fig. 2). Soma FI profiles for all motif-targeted groups were similar to the control except the Nav1.6-motif group which had significantly lower FI (p<0.001; Fig. 2A). Dendrite FI profiles for both Kv2.1- and Nav1.6-targeted groups were significantly different than the control (Kv2.1-motif: p<0.001; Nav1.6-motif: p<0.001; Fig. 2B) while there was no difference in the somatodendritic-targeting motifs. Axon FI profiles for both Kv2.1- and Nav1.6-targeted groups were again significantly lower than the control (Kv2.1-motif: p<0.001; Nav1.6-motif: p<0.001) along with two somatodendritic-targeting motifs: MLPH-motif (p<0.001) and NLG1-motif (p<0.001) Fig. 2C).

### Suitability of Motifs for Center- or Surround-targeting

For creating center or surround receptive field in RGCs, motifs that produce a differential targeted expression to the center and surround of the dendritic field would be required. In addition, motifs that decrease axonal expression are preferred since ChR2-expressing axons of RGCs may directly generate action potentials by light and thus interfere with the retinotopic mapping in higher visual centers. Although both center-targeting motifs, the Kv2.1- and the Nav1.6-motif, had significantly lower dendritic and axonal (beyond the AIS) expression compared to the control, the Nav1.6-motif had significantly lower soma expression. Therefore, the Kv2.1-motif was selected to be investigated further for center targeting. For the surround-targeting motifs, while both the MLPH-motif and the NLG1-motif had significantly lower expression in the axon compared to control, the NLG1-motif was selected for surround targeting because the MLPH-motif resulted in intracellular inclusions. Note that although surround-targeted motif expression was observed in the entire dendritic tree and soma which would overlap with the center-targeted zone, an antagonistic center-surround receptive field could still be generated as long as net excitatory and inhibitory zones are produced (see Discussion).

### Morphological Dendritic Field Size of RGCs with Targeted Motifs

**Figure 3:**
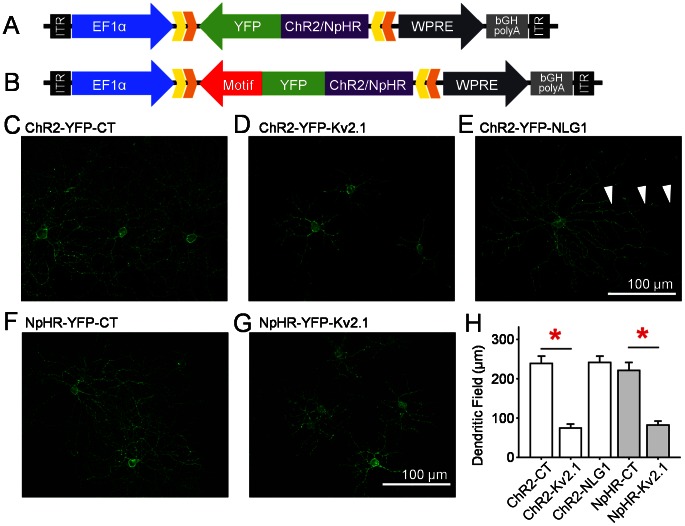
Cre-dependent viral constructs and expression in Pcp2-cre RGCs. A, B) The control AAV2/2-DIO construct carrying ChR2- or NpHR-YFP gene (A) and the modified construct with targeting-motif inserted in the 3′ end of YFP are driven by the ubiquitous Ef1α promoter (B). C–E) PCP2-RGC expression of the ChR2-YFP (C) without motif targeting (control), with (D) Kv2.1-motif targeting and with (E) NLG1-motif targeting. F, G) PCP2-RGC expression of the NpHR-YFP (F) without motif targeting (control) (G) and with Kv2.1-motif targeting. H) Quantitative comparison of the dendritic field sizes of ChR2-YFP and NpHR-YFP expressing PCP2-RGCs. In the targeted ChR2-YFP group, dendritic field expression diameter in PCP2-RGCs for the Kv2.1-motif (75±5 µm; n = 33) and NLG1-motif (242±8 µm; n = 27) were compared to the control (239±9 µm; n = 30). The Kv2.1-motif resulted in a significantly different targeted dendritic field size (*p<0.001) while the NLG1-motif did not significantly differ compared to control. In the NpHR-YFP group, targeted dendritic field diameter in PCP2-RGCs for the Kv2.1-motif (82±5 µm; n = 23) were compared to the control (221±10 µm; n = 23). The Kv2.1-motif resulted in a significantly different targeted dendritic field size (*p<0.001).

To evaluate the RGC morphological dendritic field size and the ChR2 or NpHR-mediated light response properties after center-targeting with the Kv2.1-motif and surround-targeting with the NLG1-motif, we used a transgenic Cre mouse line, Tg(Pcp2-cre)1Amc/J (referred to as Pcp2-cre), in which the expression of Cre is limited to a few RGC subtypes (referred to as PCP2-RGCs) in additional to bipolar cells [27]. Limiting the study to a small population of RGCs allowed for better visualization of individual RGCs. In addition, it reduced the amount of variance in the results regarding dendritic field sizes and physiological properties of RGCs. The expression of ChR2-YFP and NpHR-YFP with or without motifs in RGCs was achieved by using cre-dependent rAAV2 vectors driven by the neuronal promoter, EFlα (Fig. 3A and B). When injected intravitreally, the vectors are able to infect Cre-expressing RGCs but not bipolar cells [27]. The expression of ChR2-YFP or NpHR-YFP with Kv2.1-motif resulted in strong targeting of the proximal dendrites in addition to the soma in the PCP2-RGCs (Fig. 3D and G). Thus, the dendritic field expressing ChR2-YFP or NpHR-YFP with Kv2.1-motif is dramatically reduced compared to control (Fig. 3C and F). On the other hand, the expression of ChR2-YFP with NLG1-motif extended throughout the entire dendritic tree in the PCP2-RGCs (Fig. 3E) similar to that observed in the control groups (Fig. 3C), but again the expression in the axons (indicated by arrowheads in Fig. 3E) is reduced. The anatomical dendritic field sizes for the targeted and control groups were quantitatively measured (Fig. 3H). In the ChR2-YFP group, targeted dendritic field diameter in PCP2-RGCs for the Kv2.1-motif, NLG1-motif, and control were 75±5 µm (n = 33), 242±8 µm (n = 27), and 239±9 µm (n = 30), respectively. The Kv2.1-motif resulted in a significantly different targeted dendritic field size (p<0.001) while the NLG1-motif did not significantly differ compared to control. Similarly, in the NpHR-YFP group, targeted dendritic field diameter in for the Kv2.1-motif and control were 82±5 µm (n = 23) and 221±10 µm (n = 23), respectively. Again, the Kv2.1-motif resulted in a significantly different targeted dendritic field size (p<0.001).

**Figure 4:**
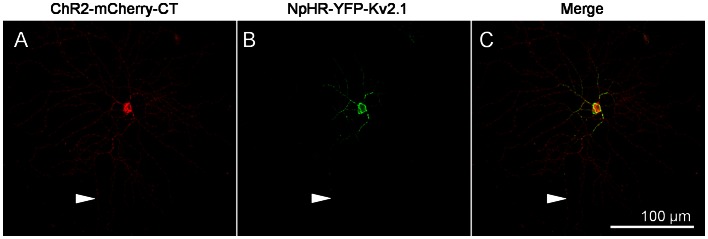
Co-expression of ChR2-mCherry and NpHR-YFP-Kv2.1. A–C) Eyes were co-injected with virus vector carrying ChR2-mCherry and NpHR-YFP-Kv2.1. The images of ChR2-mCherry (red; A) and NpHR-YFP-Kv2.1 (green; B) were merged to demonstrate a center-surround type organization in the dendritic field of a single RGC (C). The axon is indicated by arrowheads.

Central targeting with the Kv2.1-motif can be more clearly visualized in a RGC infected by two viral vectors carrying ChR2-mCherry without motif and NpHR-YFP with the Kv2.1-motif (Fig. 4). Note the reduced axonal expression with the Kv2.1-motif (indicated by arrowheads).

### Physiological Response Field Size of RGCs with Targeted Motifs

Since the Kv2.1-targeted-dendritic field is significantly smaller than the surround, we went on to examine how this targeting affected the ChR2- and NpHR-mediated response field size in PCP2-RGCs. The response field size was assessed by MEA recordings in retinal whole-mounts. All recordings were made under the condition that intrinsic photoreceptor light responses were blocked with L-AP4 (10 µM) and CNQX (6-cyano-7-nitroquinoxaline-2,3-dione) (25 µM) to isolate ChR2- or NpHR-mediated activity.

**Figure 5:**
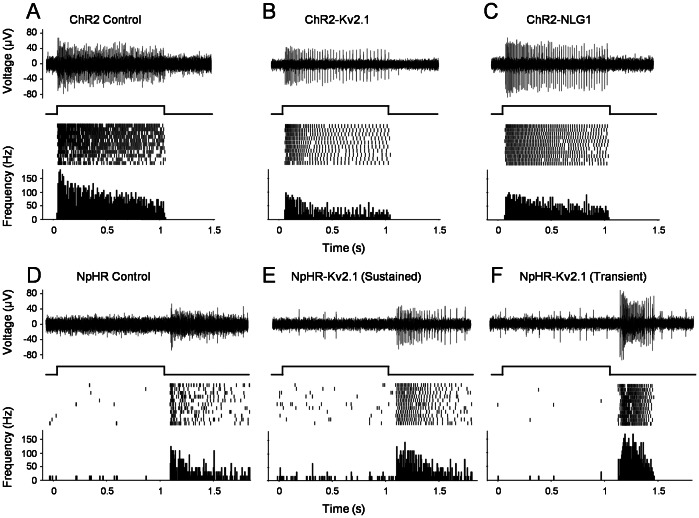
MEA recordings of ChR2 and NpHR-mediated spike responses of RGCs to whole-field light stimulation with and without motifs. A–C) ChR2-mediated light response without motif (A), with Kv2.1 (B) and NLG1 motif (C). D–F) NpHR-mediated light responses without motif (D) and with Kv2.1 motif that displayed sustained (E) or transient (F) OFF responses. In each panel, a single trace of light-evoked spike activity, a raster plot of 10 consecutive recordings, and an averaged spike rate histogram are shown in the top, middle, and bottom panel, respectively. The responses were elicited by the combination of a blue and a green laser with the light intensity of 6×10^15^ and 1×10^16^ photons/cm^2^ sec, respectively.

The ChR2- or NpHR-mediated response properties were first confirmed with whole-field stimulation. For ChR2-expressing retinas with the Kv2.1- or NGL1-motif, sustained spiking was elicited during light stimulation (Fig. 5B and C); while for NpHR-expressing retinas with the Kv2.1-motif, sustain or transient spiking was elicited after termination of light stimulation from targeted RGCs (Fig. 5E and F). These properties are similar to that of control for ChR2 (Fig. 5A) and NpHR (Fig. 5D) without targeted motifs as previously reported [1], [8].

**Figure 6:**
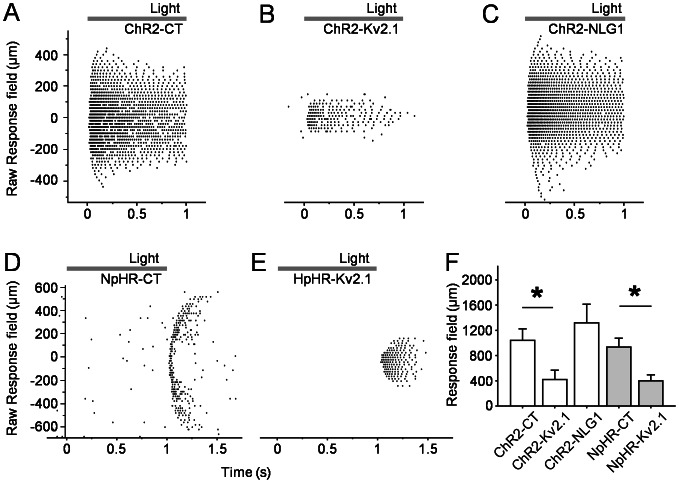
Motif-targeted response field size. A–C) Sample MEA recording traces of ChR2-YFP (A), ChR2-YFP-NLG1-motif (B), ChR2-YFPKv2.1-motif (C), NpHR-YFP (D) NpHR-YFP-Kv2.1-motif (E) expressing PCP2-RGC to a 200 µm light bar stimulus stepping in 20 µm increments. Each row of dots represent spiking activity elicited by a 1s light pulse and sequential rows represent sequential steps through the RGC receptive field. F) Quantitative comparison of the response field sizes of ChR2-YFP and NpHR-YFP expressing PCP2-RGCs. For each RGC, the physiological receptive field size was estimated from the number of steps that elicited light-driven spiking activity. The ChR2-mediated receptive field sizes for the ChR2-YFP control, Kv2.1-motif, NLG1-motif were was 1040±92 µm (n = 13), 420±74 µm (n = 11), and 1317±148 µm (n = 12), respectively. When compared to the ChR2-YFP-control, the ChR2-YFP-Kv2.1-motif resulted in a significantly different receptive field size (*p<0.0001) while the ChR2-YFP-NLG1-motif did not show a difference. Similarly, the NpHR-mediated receptive field size for the NpHR-YFP control was 935±70 µm (n = 11) and for the NpHR-YFP-Kv2.1-motif was 400±48 µm (n = 10). The Kv2.1-motif resulted in a significantly different physiological response field size compared to the control (*p<0.0001). The responses were elicited by the combination of a blue and a green laser with the light intensity of 6×10^15^ and 1×10^16^ photons/cm^2^ sec, respectively.

The response field size was then estimated by stepping a bar of light approximately 200 µm wide and 6000 µm long at 20 µm increments through the RGC response field. For ChR2-expressing RGCs, as the bar of light was stepped closer to the center of the RGC receptive field, spiking activity initiated more rapidly after light onset along with increasing spike frequency. Conversely, as the bar of light was stepped away from the receptive field center, spike activity initiation was more delayed and spike frequency decreased (Fig. 6A–C). Similar phenomena were also observed for NpHR-expressing RGCs, except the spiking activity initiated after light offset (Fig. 6D and E). For each RGC, the physiological response field size was estimated from the number of steps that elicited light-driven spiking activity and compared between the motif-targeted groups and the control. The ChR2-mediated response field size for the ChR2-YFP control was 1040±92 µm (n = 13), for the ChR2-YFP-Kv2.1-motif was 420±74 µm (n = 11), and for the ChR2-YFP-NLG1-motif was 1317±148 µm (n = 12). When compared to the ChR2-YFP-control, the ChR2-YFP-Kv2.1-motif resulted in a significantly different response field size (p<0.0001) while the ChR2-YFP-NLG1-motif did not show a difference (Fig. 6F). Similarly, the NpHR-mediated response field size for the NpHR-YFP control was 935±70 µm (n = 11) and for the NpHR-YFP-Kv2.1-motif was 400±48 µm (n = 10). Again, the Kv2.1-motif resulted in a significantly different physiological response field size compared to the control (p<0.0001; Fig. 6F).

## Discussion

### Comparison of Results to Early Studies

In this study we examined the in vivo expression profiles of eight protein targeting motifs in RGCs through rAAV-mediated delivery. Four of these motifs, Nav1.6, Kv2.1, MLPH, and NLG1, were found to produce polarized expression in RGCs. Nav1.6- and Kv2.1-motifs produced central polarization; the Nav1.6-motif targeted expression to the soma and AIS while the Kv2.1-motif targeted expression to the soma and proximal dendrites. The other two motifs, MLPH and NLG1, were broadly targeted to the somatodendritic region. All of them displayed significantly reduced expression in the axon (beyond AIS for Nav1.6). These polarized expression properties are similar to previously reported results in vitro in pyramidal cells of the hippocampus and cortex [12], [13], [16], [17], [19].

On the other hand, the remaining four motifs, AMPAR, Kv4.2, nAChR, and TLCN, did not show polarized expression in RGCs. Although these four motifs were previously reported to targeted the somatodendritic region with reduced expression in axons [14], [15], [18], [20], our results showed that expression did not differ from the control. Since most of these previous studies were examined in cultured pyramidal neurons of the hippocampus or cortex by chemical-based transfection, with except of the TLCN motif which was examined in vivo in the cerebellar Purkinje cells in transgenic mice [20], it remains unclear if the lack of polarized expression in RGCs is due to the difference in delivery methods or cell types. It’s worth nothing that although RGCs contain multiple subtypes, we did not notice a heterogeneous expression for any of the motifs among RGCs. It would be interesting to examine these motif-targeted rAAV vectors in other neurons of the brain in future studies.

### The Suitability of Motifs for Creating Center-surround Receptive Field

For the purpose of creating the center-surround receptive field, our results showed that the Nav1.6-motif is less suitable for central targeting compared to Kv2.1 because the Nav1.6-motif displayed significantly lower somatic expression (see Figure 2A). In addition, since the targeted expression of ChR2-GFP or NpHR-GFP with the Nav1.6-motif to the AIS is due to binding of the Nav1.6-motif to Ankyrin-G, this could potentially disrupt Na^+^ channel clustering at the AIS because they may compete with the native Na^+^ channels for Ankyrin-G binding [28]. Indeed, a recent study reported that targeting ChR2 to the AIS of pyramidal neurons with the Na^+^ channel motif was ineffective for evoking action potentials [29]. On the other hand, expression with the Kv2.1-motif was localized to the soma as well as the proximal dendrites roughly covering about 1/3 of the overall dendritic field. The expression of ChR2 or NpHR with the Kv2.1-motif was not observed to affect the spike firing properties of RGCs. Furthermore, our physiological recordings of ChR2 or NpHR-mediated response fields showed that the receptive field size with Kv2.1-motif targeting was markedly reduced.

For surround targeting, although both MLPH- and NGL1-motifs displayed polarized expression to the somatodendritic region with reduced axonal expression, our results indicated that the NLG1-motif is better suited for generating surround receptive field in RGCs. This is because the MLPH-motif was found to result in intracellular inclusions in cell somas, suggesting that the transgene expression with the MLPH-motif may result in certain protein trafficking problem to the plasma membrane. Furthermore, our physiological recordings also confirmed the suitability of using NGL1-motif for surround targeting because its response field size is much larger than that with central targeting. The overall response field size with the NGL1-motif appeared to be slight larger than that of the control although it is not statistically different. Thus, our results demonstrated through morphological measurements and physiological recordings that the Kv2.1- and NLG1-motifs are suitable for generating a smaller center receptive field and a larger surround receptive field through rAAV-mediated gene delivery, respectively.

Recently two motifs associated with ankyrin-G (2512 bp) and PSD (2235 bp) have been reported to create center and surround receptive fields, respectively, in RGCs by biolistic particle delivery [10]. Whether these two motifs could be suitable for rAAV-mediated delivery, however, remains to be determined.

### Comparison between Morphological Dendritic Field and Physiological Response Field

It is well known that in general the overall receptive field of a retinal ganglion cells is much larger than the size of its dendritic field [30]. The center of the receptive field is close to or slightly larger than the size of the dendritic filed [31]. This is because the surround receptive field is generated by lateral inhibition of interneurons, horizontal and/or amacrine cells [32], [33]. Interestingly, the estimated physiological response field size measured by ChR2 light activation in the PCP2-RGC was greater than its respective morphological dendritic field size (comparing Fig. 3H and Fig. 6F). Even after accounting for the over-estimated response field due to the 200 µm bar stepping in 20 µm increments, response field sizes are still about 4–5 times greater than dendritic field sizes of the respective groups. This was not due to the off targeting of ChR2 and NpHR to presynaptic neurons, such as amacrine and horizontal cells because no Cre positive amacrine cells or horizontal cells were present in the Pcp2-cre mouse line [27], [34]. The enlarged physiological response field measurements could be due to scattering of the light beam as it passed through the MEA recording chamber and retinal tissue and/or the reflection of the light stimulus off the white mounting filter paper. In particular, the light stimulation intensity used for the MEA recordings was estimated to be ∼10 times above threshold activation levels for the ChR2-GFP expressing RGCs. Above threshold light stimuli have been reported to result in enlarged estimation of receptive field size in the normal retina [35]. Also, because we used a 200 µm wide stimulus bar to map the receptive field size, this limited the resolution of the resulting receptive estimate since the width of the receptive field estimate represents the convolution of the actual receptive field width with the stimulus. We considered the possibility that the measured spatial extents reflect the overall light sensitivity produced by the different motifs, rather than the spatial extents of the dendritic arbors. However, this seems very unlikely because the NpHR-expressing RGCs are about a log unit less sensitive than ChR2-expressing RGCs. If it's a sensitivity issue, the response field size of NpHR-expressing RGCs would be expected to be significantly smaller compared to ChR2-expressing groups; but that's not the case. The exact factors contributing to the dendritic field and response field size discrepancy remains to be investigated.

It should also be mentioned that the transgenic Cre mouse line, Pcp2-cre, used in this study contain five morphologically different RGCs with average dendritic field sizes ranging from 155 µm to 295 µm although the majority of these GCs belong to two subtypes with the average dendritic field size of 219 µm and 155 µm, respectively [34]. The expression of light sensors in these mixed subtypes of PCP2-RGCs certainly affected the accuracy of our data analysis, but should not alter the overall conclusion of this study. This is because the average targeted dendritic field size with the center targeting motif of Kv2.1 (75–82 µm) is much smaller the average dendritic field size of the smallest PCP2-RGCs (155 µm).

Further developments for implementation of artificial RGC center-surround antagonism.

The ability for rAAV-mediated differential targeting of optogenetic tools to the center and surround regions of the RGCs is proof of principal for the restoration of center and surround antagonistic receptive fields in RGCs in vivo. Thus, these motifs could be potentially useful in therapeutic applications. However, several issues need to be taken into consideration and further development will be needed toward this purpose. First, since the center region is overlapped by both center and surround targeting motifs, in order to create a net ON or OFF center, expression of the depolarizing and hyperpolarizing light sensors in RGCs need to be balanced. This balance would need to take into account the protein expression level, light sensitivity, and spectral sensitivity of both light sensors. The use of a pure encompassing surround targeting motif may in part circumvent the problem, but such a motif has yet to be identified. Second, the development of more light sensitive hyperpolarizing light sensors is needed. This is because the light sensitivity of NpHR is much lower than that of ChR2. The light sensitivity of NpHR-expressing RGCs is about one log unit lower than that of ChR2-expressing RGCs [8]. Recently a number of more light sensitive ChR mutants have been reported [36]–[39]. In contrast, few hyperpolarizing light sensors are available [36]. ArchT, a light-driven outward proton pump, has been recently reported to be more sensitive than NpHR [40], although the expression and light response properties have not yet been examined in retinal neurons. In addition, because the peak wavelength sensitivity of NpHR is around 570 nm while the wild-type ChR2 is around 460 nm, the use of red-shifted ChR variants would be preferred [38], [39]. Furthermore, in order to restore the center-surround receptive field to mimic intrinsic ON and OFF pathways [41], the depolarizing and hyperpolarizing light sensors need to be targeted to ON and OFF RGCs, respectively. Currently, ON and OFF RGC specific promoters have not yet been discovered. Nevertheless, before ON and OFF RGC specific promoters are found, using a ubiquitous promoter to convert all RGCs into ON-center or OFF-center RGCs may still restore certain useful vision [42]. For the latter, the use of targeted center-surround expression would be expected to increase spatial sensitivity.

### Beyond the Retina

One advantage of targeting motifs is that their applications are not limited to the study of retinal neurons. It is conceivable that targeting motifs can be linked to a wide variety of fluorescent proteins to visualize neuron morphology with a new level of localization detail. Targeting motifs could also be linked with other neuromodulating proteins to manipulate neuron physiology. The subcellular targeting motifs suitable for rAAV-mediated transgene delivery, especially of optogenetic tools as demonstrated in this study, could also be a valuable tool for precise protein targeting for studying physiological function and clinical applications in other areas of the central nervous system.
